# Crystallographic and Thermodynamic Evidence of Negative Coupling in the Flavin-Dependent Tryptophan Halogenases AbeH and BorH

**DOI:** 10.1021/acsomega.4c09590

**Published:** 2025-01-08

**Authors:** Md Ashaduzzaman, Kazi Lingkon, Aravinda J. De Silva, John J. Bellizzi

**Affiliations:** Department of Chemistry and Biochemistry, The University of Toledo, Toledo, Ohio 43606, United States

## Abstract

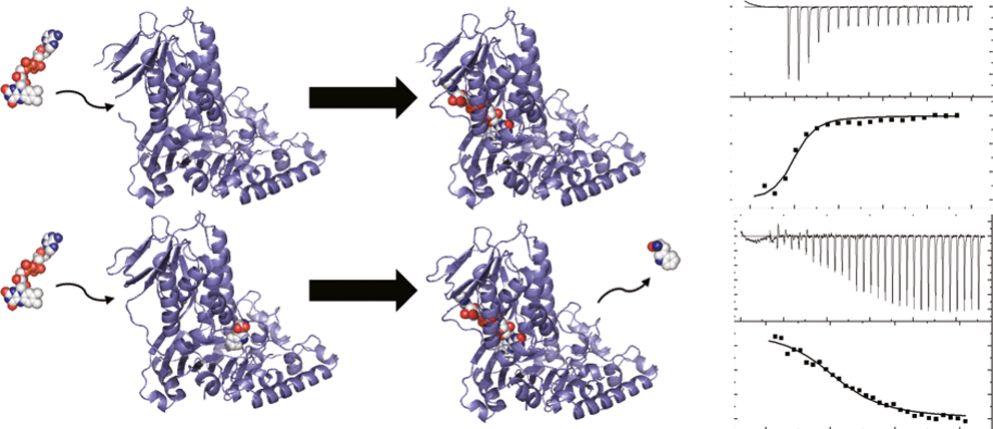

Flavin-dependent halogenases (FDHs) regioselectively halogenate aromatic substrates using halide ions, O_2_, and reduced flavin (FADH_2_) at physiological temperatures in aqueous solution, making them a green alternative to conventional synthetic methods for aryl halide preparation. To better understand mechanistic details that limit FDH catalytic efficiency and potentially hinder their application as *in vitro* biocatalysts, we investigated the halogenation activity, substrate scope, crystal structures, and ligand binding of the Trp-5-halogenase AbeH and the Trp-6-halogenase BorH. Partitioning of FAD and Trp into different subunits of BorH crystals and an inability to incorporate Trp into AbeH/FAD crystals suggested that binding of flavin and Trp are negatively coupled in both proteins. Isothermal titration calorimetry and fluorescence quenching experiments confirmed that both AbeH and BorH formed binary complexes with FAD or Trp, but Trp could not form ternary complexes with preincubated AbeH/FAD or BorH/FAD complexes. FAD could not bind to BorH/Trp complexes, but FAD appears to displace Trp from AbeH/Trp complexes in an endothermic entropically driven process. Observation of negative coupling in halogenases from two different clades with topological differences in their substrate binding sites suggests that this property and the limitations it places on catalytic efficiency may be a general characteristic of the FDH family.

## Introduction

Flavin-dependent halogenases (FDHs; EC 1.14.19.9, 1.14.19.58, 1.14.19.59; Interpro IPR006905; Pfam PF04820) install chlorine and bromine in natural products produced by bacteria and fungi from terrestrial and marine environments.^1−4^ Examples of FDH-generated halogenated secondary metabolites with potent biological activity are rebeccamycin,^5,6^ chloramphenicol,^7,8^ and pyoluteorin.^9,10^ FDHs have attracted attention because, in contrast to common synthetic methods for aryl halide preparation by electrophilic aromatic substitution (EAS),^11^ they use a safe and inexpensive halogen source (halide salts), operate in aqueous solution at ambient temperature, and demonstrate precise regioselectivity that can override electronic directing effects.^12−14^ For this reason, there is great interest in the development of FDHs as biocatalytic tools^14−16^ for green organic synthesis of halogenated end products^17^ and aryl halides for use in transition metal catalyzed cross coupling reactions.^18−20^

Many of the best-characterized FDHs regioselectively halogenate l-tryptophan (Trp), a common building block for diverse natural products.^5,21−24^ The FDH reaction mechanism (originally proposed for the Trp-7-halogenase PrnA^25^) starts with the reaction of reduced flavin (FADH_2_) and O_2_ to generate a C4a-hydroperoxyflavin intermediate, as occurs in flavin-dependent monooxygenases.^26,27^ The halogenase mechanism then diverges from the monooxygenase mechanism, because instead of reacting directly with an aromatic substrate to form a C–O bond, the hydroperoxyflavin intermediate reacts with a bound Cl^–^ ion to form HOCl and hydroxyflavin.^28^ HOCl generation occurs >10 Å away from where the Trp (or other aromatic substrate) binds, and the HOCl must diffuse through a tunnel toward a conserved Lys,^29^ which either hydrogen bonds with HOCl and positions the Cl for reaction with Trp by EAS^25,30,31^ or reacts with HOCl to form a chloramine, which in turn transfers Cl to Trp by EAS.^32,33^ Regioselectivity is dictated by the orientation of the Trp (or other aromatic substrate) in the binding pocket, with the atom closest to the conserved Lys receiving the halogen atom.^25,30,31^ Most characterized FDHs can brominate as well as chlorinate and can halogenate other aromatic compounds besides their native substrate (though often with lower catalytic efficiency), but the precise set of substrates and regioselectivity varies from one to another.^23,27,34−36^

Unlike most flavoproteins, FDHs do not bind flavin as a prosthetic group or catalyze the reduction of FAD back to FADH_2_. FDHs belong to the two-component diffusible-flavin monooxygenase family,^26^ in which FADH_2_/FAD act as cosubstrate and coproduct for an oxidoreductase that relies on a partner flavin reductase enzyme which reduces FAD to FADH_2_ using NAD(P)H.^22^ Flavin must be released into solution and diffuse back and forth between the two subunits to complete the catalytic cycle, creating the potential for unproductive side reactions and contributing to catalytic inefficiency.

Obstacles to synthetic applications of FDHs include low catalytic efficiency,^14,24,28^ suboptimal stability,^37^ and limited substrate scope.^15^ Techniques applied to expand substrate scope, improve thermostability and catalytic lifetime, and optimize reaction rate have included formation of supramolecular assemblies,^18,38−41^ cofactor reduction systems and novel reductants,^17,42,43^ molecular dynamics simulation,^28,44−47^ site-directed mutagenesis,^20,29,31,48−51^ and directed evolution^52−54^ of FDHs. The number of known FDHs has steadily increased through targeted discovery of new enzymes with different halide preferences,^36,55,56^ substrate scopes,^36,55,57−60^ regioselectivities,^61^ and thermostabilities^23,24^ via experimental and bioinformatic prospecting of microbial genomes and metagenomes from diverse ecological niches.^4,34,35,62,63^

**Figure 1:**
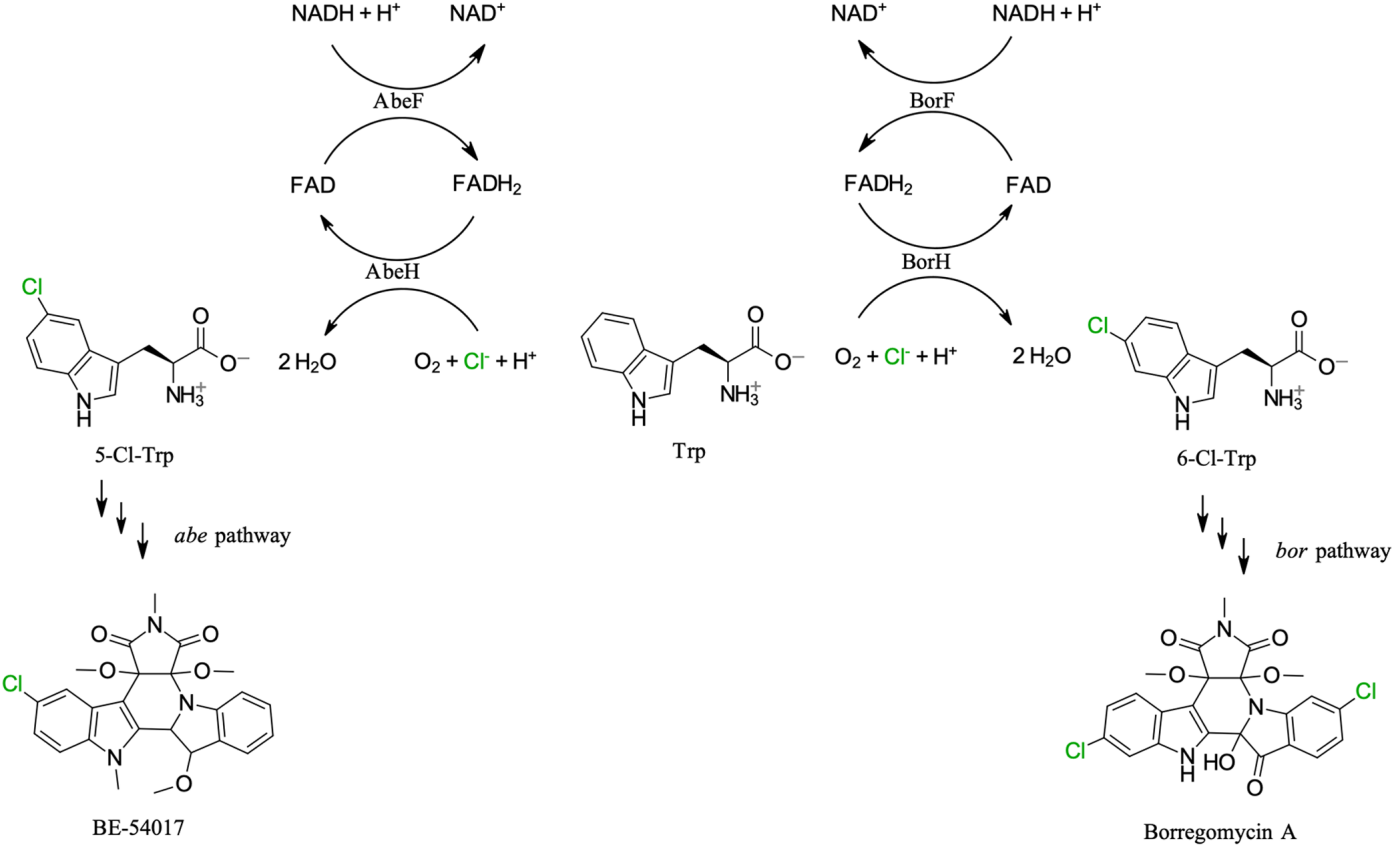
AbeH and BorH are flavin-dependent tryptophan halogenases. AbeH uses FADH_2_, O_2_, and Cl^–^ to convert Trp to 5-Cl-Trp and AbeF reduces the FAD released by AbeH to FADH_2_ using NADH to complete the catalytic cycle. Similarly, BorH uses FADH_2_ generated by BorF to convert Trp to 6-Cl-Trp. The other enzymes encoded by the *abe* and *bor* gene clusters convert 5-Cl-Trp and 6-Cl-Trp to the bisindole alkaloids BE-54017 and borregomycin A, respectively.

To this end, we have investigated the structures and halogenation reactions of the FDHs AbeH and BorH to add them to the existing catalytic toolbox. AbeH (UniProtKB F6LWA5) and BorH (UniProtKB M9QSI0) are FDHs produced by uncultured soil actinomycetes and encoded in gene clusters responsible for the biosynthesis of the chlorinated indolotryptoline natural products BE-54017 (*abe*: GenBank JF439215)^64^ and borregomycin A (*bor*: GenBank AGI62217).^65^ These natural products and the gene clusters responsible for their synthesis were discovered from environmental DNA libraries collected from Anza-Borrego desert soil.^64,65^ Based on their sequence homology to known FDHs and the positions of Cl in BE-54017 and borregomycin A, which are assembled by oxidative dimerization of Trp, AbeH was hypothesized to be a Trp-5-halogenase and BorH was predicted to be a Trp-6-halogenase^3,64,65^ (Figure 1). The *abe* and *bor* gene clusters each contain an open reading frame predicted to encode a short chain flavin reductase (FR; EC 1.5.1.36; InterPro IPR002563; Pfam PF01613), and it was proposed that AbeF (UniProtKB F6LWA7) and BorF (UniProtKB M9QXS1) were the FRs responsible for supplying AbeH and BorH, respectively with FADH_2_ (Figure 1).

We have previously experimentally verified that BorF and AbeF are FRs that catalyze the NADH-dependent reduction of FAD to FADH_2_ and shown that BorH can not only brominate and chlorinate Trp at C6 as predicted, but can also halogenate anthranilamide, 6-aminoquinoline, kynurenine, tryptamine, and 3-indolepropionic acid.^23,66^ We also solved the X-ray crystal structure of BorH/Trp (6UL2)^23^ to reveal Trp bound in the same orientation previously seen in the structure of the Trp-6-FDH Thal,^31,67^ reinforcing the prevailing model that substrate orientation provides the structural basis for regioselectivity in FDHs.

In this paper, we report *in vitro* characterization of the Trp-5-halogenase activity of AbeH, the crystal structures of AbeH with and without bound FAD, and crystal structures of apo-BorH along with BorH in complexes with FAD, Trp, and 6-Cl-Trp. Despite extensive efforts, we were unsuccessful in obtaining crystal structures of ternary FDH/FAD/Trp complexes for either AbeH or BorH, though such complexes have been previously reported for RebH,^68^ PrnA,^25^ and PyrH.^30^ Our inability to solve the ternary complex structures, along with previous reports of negative coupling of flavin and Trp binding in RebH^68^ and Thal^67^ motivated us to investigate the binding of FAD and Trp to AbeH and BorH using isothermal titration calorimetry (ITC) and fluorescence quenching. Our results show that flavin cosubstrate/coproduct (FADH_2_/FAD) and substrate (Trp) cannot bind simultaneously to either AbeH or BorH. A structural requirement that flavin dissociates from the enzyme after HOCl formation but before Trp binding likely represents a limiting factor for the catalytic efficiency of both enzymes. Further investigation of this negative coupling could lead to the identification of the structural basis for communication between the two binding sites and facilitate the design of more efficient mutant FDHs unhindered by negative coupling.

## Results

### AbeH is a Tryptophan-5-halogenase

AbeH with an N-terminal His_6_-tag was overexpressed in *Escherichia coli* and purified to homogeneity by immobilized metal affinity, anion exchange, and size exclusion chromatography (SEC) (Figure S1). AbeH eluted from SEC as an apparent monomer. Purified AbeH converted Trp to Cl-Trp in the presence of AbeF, FAD, NADH, and NaCl (Figure S2) as determined by reversed phase high-performance liquid chromatography (RP-HPLC) analysis of reaction mixtures monitoring A_280_ for disappearance of Trp (*t*_R_ = 10.3 min) and appearance of a new peak (*t*_R_ = 11.3 min) with the same retention as a 5-Cl-Trp standard (Figure S2). Electrospray ionization mass spectrometry (ESI-MS) analysis of this peak (*m*/*z*: 239.0574 and 241.0559; 3:1 ratio) confirmed the product was monochlorinated Trp (Table S1). ^1^H NMR verified that the isomer produced by AbeH is 5-Cl-Trp, verifying the predicted regioselectivity based on the position of the Cl in BE-54017 (Figure S3). Substituting NaBr for NaCl resulted in suppression of the 5-Cl-Trp peak and the appearance of a later-eluting peak (*t*_R_ = 11.5 min) matching the retention time of a 5-Br-Trp standard (Figure S2), and the identity of this product was confirmed as monobrominated Trp by ESI-MS (*m*/*z*: 283.0103, 285.0078; 1:1 ratio). No product peak was observed when NaCl was replaced with NaI. The specific activity of AbeH for chlorination of Trp is approximately 2.5× the specific activity for Trp bromination (87 and 34 μM min^–1^ mg^–1^, respectively) (Figure S4).

### AbeH and BorH Can Chlorinate and Brominate Indole, Benzene, and Quinoline Derivatives

We tested the halogenation activity of AbeH using 20 additional aromatic substrates and found that AbeH could chlorinate and brominate compounds with phenyl and quinoline scaffolds as well as indoles (Table 1, Table S1, and Figure S5). AbeH did not halogenate 5-cyanoindole, 5-hydroxytryptophan, and serotonin, possibly because the C5 position was already substituted. Despite having an unsubstituted C5, tryptamine was not halogenated by AbeH, though 3-indolepropionic acid was, which we interpreted to mean that the carboxylate of Trp (missing in tryptamine) plays a larger role in binding affinity than the amino group (missing in 3-indolepropionic acid). Indole, which lacks both the amino group and carboxylate of Trp, is efficiently chlorinated and brominated by AbeH, but indole is known to be much more reactive than Trp toward electrophilic aromatic substitution, which could lead to higher yields even if the binding affinity is lowered. AbeH halogenated anthranilamide, but not anthranilic acid, benzamide, 3-aminobenzamide, or 4-aminobenzamide, suggesting that both the amino group and amide are required, and they must be in a 1,2 orientation. Similarly, 6-aminoquinoline and 7-aminoquinoline were chlorinated and brominated by AbeH, but quinoline, 5-aminoquinoline, and 8-aminoquinoline were not. Though AbeH prefers chlorination over bromination for Trp, for all other halogenated products, the brominated product was produced in higher yield than the chlorinated product under the same reaction conditions (Table 1, Table S1, and Figure S5).

**Table 1:**
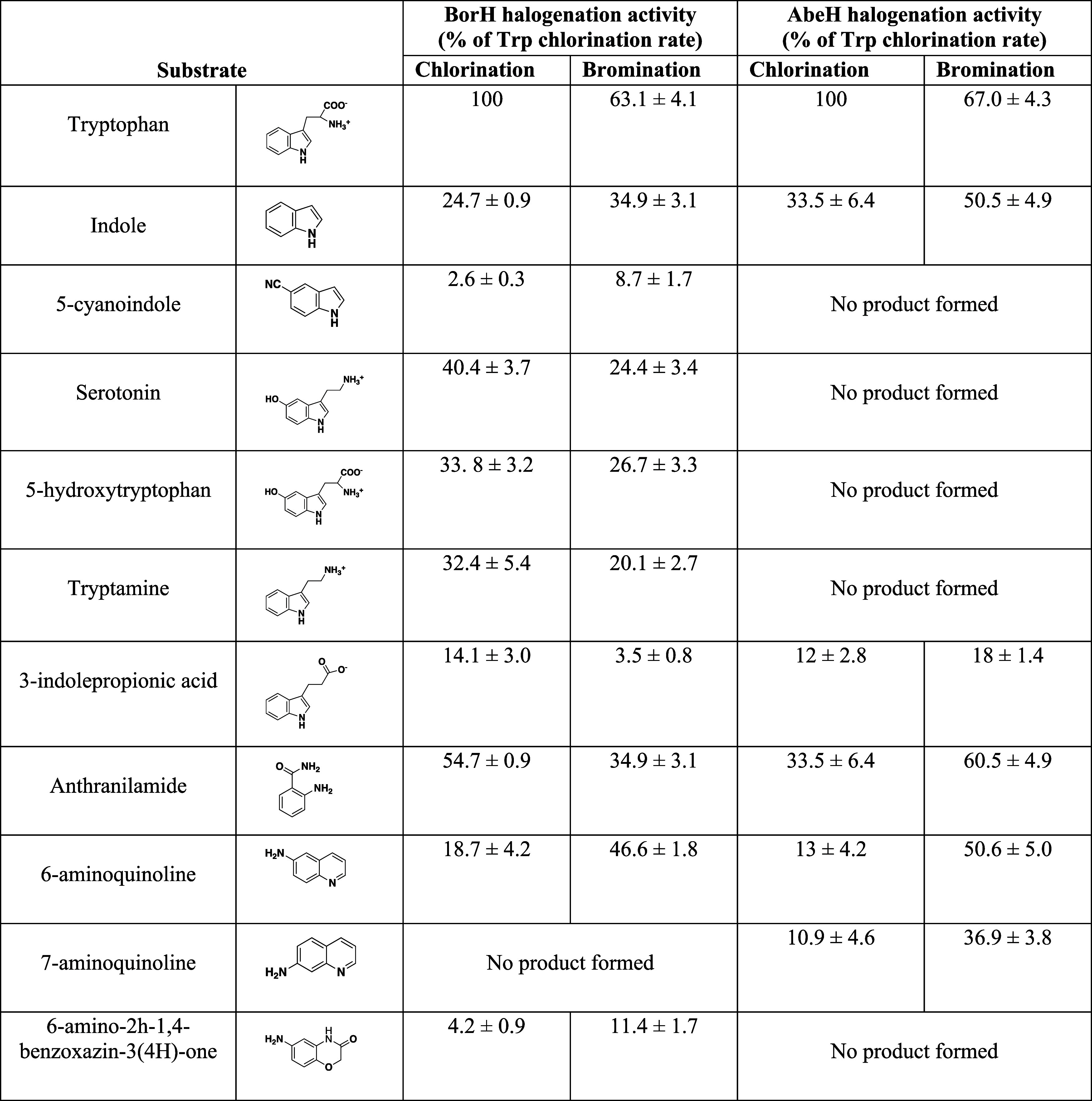
Relative Activities for Halogenation of Aromatic Substrates by BorH and AbeH

We compared BorH activity against the same set of substrates (Table 1, Table S1, and Figure S6). As we previously observed,^23^ BorH halogenated a wide range of indole derivatives as well as anthranilamide and 6-aminoquinoline, but unlike AbeH, it could not halogenate 7-aminoquinoline. BorH could halogenate 6-amino-2*H*-1,4-benzoxazin-3(4*H*)-one, which AbeH did not halogenate. BorH showed a higher rate of bromination than chlorination for indole, 5-cyanoindole, 6-aminoquinoline, and 6-amino-2*H*-1,4-benzoxazin-3(4*H*)-one, but a higher yield of chlorination than bromination for the other substrates tested.

### Crystal Structures of AbeH/FAD and Apo-AbeH

We grew diffraction-quality single crystals of AbeH in two crystal forms (Table 2). Orthorhombic AbeH crystals grew only in the presence of FAD and crystallized in space group *P2*_1_2_1_2_1_ with two AbeH/FAD complexes in the asymmetric unit (ASU). Apo-AbeH crystallized in *P2*_1_ with four molecules in the ASU.

**Table 2:** X-ray Diffraction Data and Refinement Statistics for AbeH and BorH Crystal Structures

parameters	Apo-AbeH (PDB: 8FOX)	AbeH/FAD (PDB: 8FOV)	BorH/Trp + BorH/FAD (PDB: 8TTI)	BorH/6-Cl-Trp (PDB: 8TTJ)	Apo-BorH (PDB: 8TTK)
wavelength (Å)	0.97857	0.97872	1.033	1.033	1.033
beamline	APS 21-ID-G	APS 21-ID-F	APS 21-ID-D	APS 21-ID-D	APS 21-ID-D
resolution range (Å)	60.1–1.89 (1.958–1.89)	53.91–1.86 (1.926–1.86)	54.72–1.98 (2.05–1.98)	34.98–1.98 (2.05–1.98)	37.92–1.98 (2.05–1.98)
space group	*P*2_1_	*P*2_1_2_1_2_1_	*P*2_1_	*P*2_1_	*P*2_1_
unit cell (Å, °)	63.4496, 251.13, 68.01	56.71, 112.29, 173.59	73.68, 157.41, 112.83	73.52, 157.971, 112.58	73.74, 157.58, 112.97
	90, 108.71, 90	90, 90, 90	90, 104.06, 90	90, 104.06, 90	90, 104.25, 90
total reflections	319567 (31371)	186383 (18541)	312471 (31632)	331395 (33446)	321503 (32446)
unique reflections	159987 (15847)	93637 (9276)	163198 (16589)	169702 (16993)	164806 (16702)
multiplicity	2.0 (2.0)	2.0 (2.0)	1.9 (1.9)	2.0 (2.0)	2.0 (1.9)
completeness (%)	99.88 (99.37)	99.63 (99.96)	93.91 (93.15)	98.24 (98.67)	95.03 (96.50)
mean ⟨*I*/σ(*I*)⟩	9.27 (2.88)	7.10 (2.06)	3.74 (0.62)	4.91 (1.06)	5.59 (0.98)
Wilson B-factor	18.52	23.36	30.89	27.45	26.22
*R*_merge_	0.04645 (0.274)	0.06443 (0.3797)	0.1345 (1.316)	0.997 (0.7698)	0.09669 (0.753)
*R*_meas_	0.06569 (0.3875)	0.09112 (0.537)	0.1902 (1.861)	0.1401 (1.089)	0.1367 (1.065)
*R*_pim_	0.04645 (0.274)	0.06443 (0.3797)	0.1345 (1.316)	0.09909 (0.7698)	0.09669 (0.753)
CC1/2	0.996 (0.8)	0.99 (0.708)	0.98 (0.1)	0.987 (0.247)	0.957 (0.377)
CC*	0.999 (0.943)	0.998 (0.911)	0.995 (0.426)	0.997 (0.629)	0.989 (0.74)
reflections used in refinement	159921(15845)	93623 (9274)	162282 (16029)	169634 (16965)	164625 (16680)
reflections used for *R*_free_	2001 (208)	894 (89)	1989 (198)	1996 (196)	1993 (195)
*R*_work_	0.1588(0.2149)	0.1633(0.2414)	0.2288 (0.3778)	0.1982 (0.3326)	0.2210 (0.3613)
*R*_free_	0.1834(0.2593)	0.1960(0.2634)	0.2701 (0.4186)	0.2362 (0.3864)	0.2489 (0.4160)
CC (work)	0.965 (0.896)	0.966 (0.851)	0.931 (0.457)	0.950 (0.613)	0.934 (0.678)
CC (free)	0.961 (0.853)	0.938 (0.866)	0.913 (0.328)	0.937 (0.604)	0.923 (0.617)
number of non-hydrogen atoms	17945	8945	18159	18166	17760
macromolecules	15940	7983	16745	16784	16652
ligands	60	184	171	140	80
solvent	1945	778	1243	1242	1028
protein residues	1985	995	2083	2084	2076
RMS (bonds)	0.003	0.012	0.003	0.005	0.003
RMS (angles)	0.65	1.18	0.50	0.72	0.61
Ramachandran favored (%)	97.14	96.24	97.04	97.43	96.93
Ramachandran allowed (%)	2.86	3.76	2.96	2.57	3.07
Ramachandran outliers (%)	0.00	0.00	0.00	0.00	0.00
rotamer outliers (%)	0.24	0.6	0.70	0.29	1.29
clash score	3.36	3.32	5.90	4.02	7.06
average B-factor	22.6	28.94	44.20	37.97	39.65
macromolecules	21.37	28.13	44.15	37.74	39.62
ligands	36.09	31.66	51.27	56.75	52.90
solvent	32.22	36.58	43.97	38.95	39.09
number of TLS groups	23	15	25	25	20

**Figure 2:**
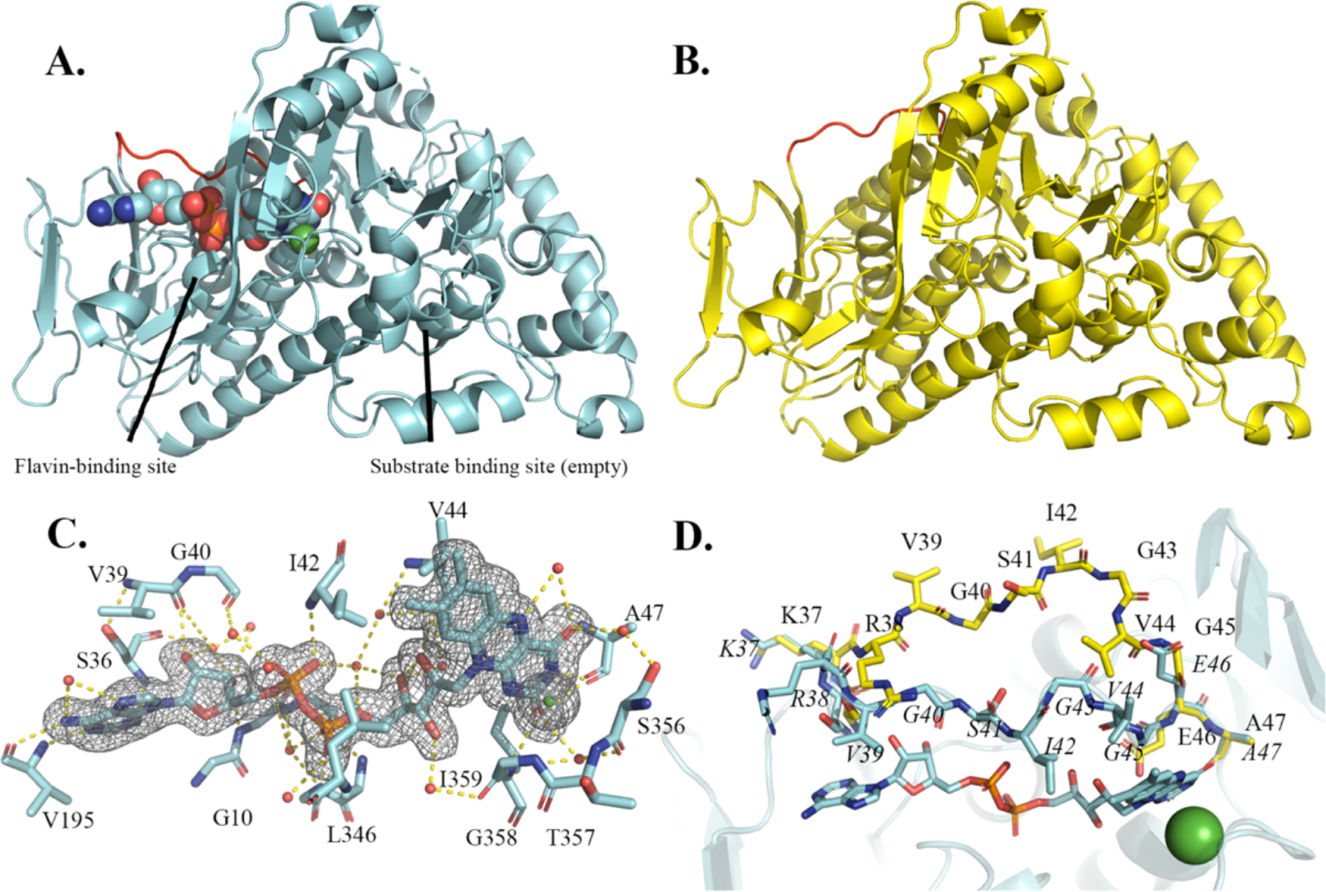
X-ray crystal structures of AbeH show two conformational states of the flavin binding loop. **A**. Ribbon diagram of AbeH/FAD (8FOV chain A; cyan). FAD is shown as a space filling model, and Cl^–^ is a green sphere. The flavin binding loop (in red) is in the closed conformation. **B.** Ribbon diagram of Apo-AbeH (8FOX chain C; yellow). The flavin binding loop (in red) is in the open conformation. **C.** Flavin binding site of AbeH/FAD (8FOV chain A; cyan). FAD and residues contacting FAD are shown as sticks, Cl^–^ as a green sphere, water molecules as red spheres, and hydrogen bonds as dashed lines. The mesh is a Polder *F*_o_ – *F*_c_ omit map contoured at 3σ with FAD and Cl^–^ omitted from the map calculation. **D.** Superposition of the FAD binding sites of apo-AbeH (8FOX chain C; yellow) and AbeH/FAD (8FOV chain A; cyan), illustrating the open and closed conformations of the flavin binding loop.

The AbeH/FAD (Figure 2A; 8FOV) structure was solved at 1.86 Å resolution using molecular replacement with Th-Hal^24^ (5LV9, 66% sequence identity to AbeH) as the search model. AbeH comprises 16 α-helices, 7 3_10_-helices, 1 π-helix and 24 β-strands, and as expected has the fold originally reported for PrnA^25^ and since seen in numerous other FDH structures,^23,30,31,47,68−70^ consisting of a box-shaped subdomain with an appended pyramidal subdomain (Figure 2A). The box has a Rossmann-like fold containing the flavin binding site and in other FDHs, the substrate binding site has been shown to lie at the interface between the box and the pyramid. Lys75 and Glu352 superimpose with the catalytic Lys and Glu residues identified in other FDHs structures. Both chains in the AbeH/FAD structure have a disordered region (Gly148-Gln160, between η3 and β9 in the box subdomain), which in other FDHs forms a lid over the Trp binding site when occupied. Chain A has a second missing segment (Asp261–Arg265). This region forms α-helix α8 between β16 and β17 in chain B. There are also two residues missing from the C- terminus of both chains. The interface between the two chains involves 42 residues from each chain and buries approximately 1610 Å^2^. The two pyramidal subdomains nest together, aligning the rectangular box-shaped subdomains approximately parallel to one another. Though AbeH is a monomer in solution according to SEC, it crystallizes with the same dimer interface as that observed in other FDH structures (some of which are reported to be monomers in solution, and some dimers^14^).

Density for FAD could be clearly seen and modeled in both chains (Figure 2C). A Cl^–^ ion was modeled in each chain in a strong spherical density peak 3.2 Å away from the *re*-face of the isoalloxazine ring of FAD (green sphere in Figure 2A,C,D); if this peak were modeled as water instead of Cl^–^, difference maps showed residual positive density. This site corresponds to the halide binding site identified in other FDHs structures.^14^ FAD binds to AbeH in the same manner seen in previously determined FDH/FAD structures. The isoalloxazine occupies a hydrophobic pocket with the *re*-face packed against η6 and the *si*-face covered by the closed conformation of the flavin binding loop (located between β2 and α2) (Figure 2C,D). FAD makes hydrogen bonds with backbone atoms of Ala47, Ser 356, Ile359 (isoalloxazine), Ala13, Ile42, Leu346 (pyrophosphate), Gly10, Gly11, Ser36, and Val195 (adenosine) and 12 water-mediated hydrogen bonds (Figure 2C). The Cl^–^ forms ion-dipole interactions with the backbone amide groups of Thr357 and Gly358 (Figure 2C).

The orthorhombic crystal used to solve the AbeH/FAD structure crystallized from a solution containing AbeH, FAD, and Trp, but there was no density observed in the Trp binding site. Data were collected on 12 more AbeH/FAD orthorhombic crystals that were either grown in the presence of Trp or soaked in Trp at concentrations from 2.5–40 mM for up to 3 days, but none of the maps generated from these crystals contained density in the Trp binding site.

The overall Cα root-mean-square deviation (rmsd) between the two chains of AbeH/FAD is 0.49 Å. The major differences between the two chains are the lack of density between β16 and β17 in chain A, and a difference in the orientation of the Val39-Gly40 peptide bond in the flavin binding loop. In chain A, the carbonyl of Val39 is pointed toward FAD and accepts a hydrogen bond from the ribose C2′–OH, whereas in chain B, the peptide bond is flipped and the amide of Gly40 donates a hydrogen bond to the ribose C2′–OH.

The apo-AbeH structure (8FOX) was solved from a 1.89 Å data set collected from a monoclinic AbeH crystal using molecular replacement with chain B of the AbeH/FAD structure as a search model (Figure 2B). There are four AbeH chains in the asymmetric unit, arranged as two dimers (B/D and A/C) with the same dimer interface seen in the AbeH/FAD structure but different crystal packing between the dimers. The monoclinic crystal used to solve the apo-AbeH structure had been soaked in Trp, but there was no electron density in the Trp binding site. The most significant backbone difference between the chains in the apo-AbeH structure and the AbeH/FAD structure is in the flavin binding loop, which is closed over FAD in both chains of the complex structure but is disordered in Chains A and B and in the open conformation in chains C and D in the apo-AbeH structure (Figure 2A,B,D). In addition, the Gly10-Gly11 peptide bond is flipped, so that in apo-AbeH the Gly10 carbonyl O is hydrogen bonded to the NH of Gly14, and in AbeH/FAD, the Gly11 N–H is engaged in a water-mediated interaction with the FAD pyrophosphoryl group (a similar flip was previously observed between the open and closed conformations of Thal^31^). In the apo-AbeH structure, all four chains are missing the η3-β9 segment, chains A, C, and D are missing the segment between β16 and β17, and chains C and D are missing five additional residues at the C-terminus. The four apo-AbeH chains have Cα rmsd values ranging from 0.33–0.51 Å with one another and from 0.78–1.11 Å with chain B of the AbeH/FAD structure (Table S2).

### Crystal Structures of Apo-BorH and BorH Substrate and Product Complexes

**Figure 3:**
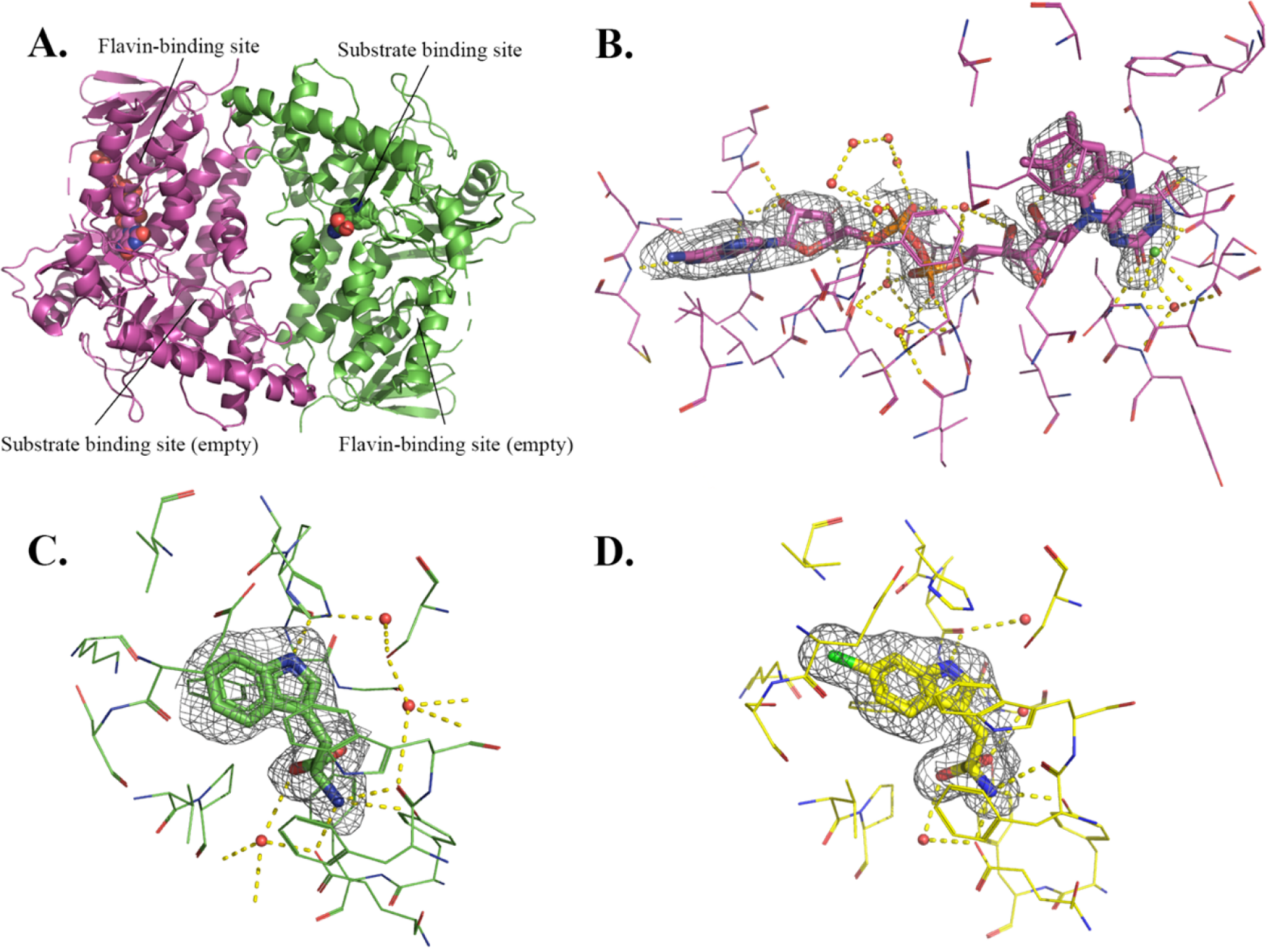
FAD and Trp partition into different BorH chains in the ASU. **A.** Ribbon diagram of crystallographic dimer of BorH/Trp (8TTI chain A; green) with BorH/FAD (8TTI chain C; magenta) from apo-BorH crystals soaked in both FAD and Trp. **B.** Flavin binding site of BorH/FAD (8TTI chain C; magenta) from the BorH/FAD + BorH/Trp crystal structure. FAD and residues contacting FAD are shown as sticks, water molecules as red spheres, and hydrogen bonds as dashed lines. The green sphere is a water molecule modeled at the position where Cl^–^ is bound in AbeH/FAD. The mesh is a Polder *F*_o_ – *F*_c_ omit map contoured at 3σ with FAD omitted from the map calculation. **C.** Trp binding site of BorH/Trp (8TTI chain A; green) from the BorH/FAD + BorH/Trp crystal structure. Trp and residues contacting Trp are shown as sticks, water molecules as red spheres, and hydrogen bonds as dashed lines. The mesh is a Polder *F*_o_ – *F*_c_ omit map contoured at 3σ with the Trp substrate omitted from the map calculation. **D.** 6-Cl-Trp bound in the Trp binding site of the BorH/6-Cl-Trp crystal structure (8TTJ chain A, yellow). 6-Cl-Trp and residues contacting Trp are shown as sticks, water molecules as red spheres, and hydrogen bonds as dashed lines. The mesh is a Polder *F*_o_ – *F*_c_ omit map contoured at 3σ with the 6-Cl-Trp product omitted from the map calculation.

We determined X-ray structures of BorH in the absence of substrates/products (apo-BorH; 8TTK), BorH complexed with product 6-Cl-Trp (BorH/6-Cl-Trp; 8TTJ), and a structure containing two BorH/FAD complexes and two BorH/Trp complexes in the asymmetric unit (BorH/Trp + BorH/FAD; 8TTI) (Figure 3 and Table 2). All structures were crystallized as apo-BorH in the same crystal form we previously used to solve the BorH/Trp complex^23^ (space group *P2*_1_ with four BorH molecules in the ASU, packed as two dimers A/D and B/C with the same dimer interface seen in the AbeH structures), and soaked with substrates/products before cryoprotection, freezing, and data collection.

In our previous BorH/Trp structure (6UL2),^23^ there was no density for the flavin binding loop in chains B–D and weak but interpretable density that allowed us to model it in the open conformation in chain A. None of the chains in the apo-BorH (8TTK), BorH/6-Cl-Trp (8TTJ), or BorH/Trp + BorH/FAD (8TTI) structures had interpretable density the flavin binding loop, even in the chains where FAD is bound. There are no significant backbone differences among the 16 chains of the four BorH crystal structures, with Cα rmsd values ranging from 0.15–0.47 Å (Table S2).

The BorH/Trp + BorH/FAD structure (8TTI) was solved from a crystal soaked in both FAD and Trp in an attempt to form a ternary complex. Chains A and B have Trp bound (but no FAD) and chains C and D have FAD bound (but no Trp) (Figure 3), arranged in the ASU as two heterodimers AD and BC (each containing one BorH/Trp and one BorH/FAD). The FAD electron density is strongest for the adenosine and phosphate groups and weaker for the ribityl and the isoalloxazine moiety. This is likely due to increased mobility of the isoalloxazine and ribityl portions of FAD due to the flavin binding loop not being closed, and mirrors previous observations for Thal,^67^ in which the AMP was modeled but no other part of FAD was seen. FAD makes hydrogen bonds with backbone atoms of Ala50, Ile361 (isoalloxazine), Ala13, Ile42, Leu348 (pyrophosphate), Gly13, Thr15, Ala16, Gly17, and Met197 (adenosine) and 12 water-mediated hydrogen bonds. Spherical density at the putative Cl^–^ binding site (adjacent to Thr359 and Gly360) was modeled as water (green sphere in Figure 3B), and unlike the AbeH/FAD structure but consistent with Thal/FAD structures,^67^ there was no residual positive density in difference maps.

The BorH/Trp interactions are unchanged when comparing the Trp bound chains (A and B) from the BorH/Trp + BorH/FAD structure to our previously determined BorH/Trp structure (6UL2).^23^ The BorH/6-Cl-Trp structure (Figure 3D) has clear density for the product in all four chains and 6-Cl-Trp makes the same interactions with BorH as those previously observed in the BorH/Trp complex. The Cl^–^ of the 6-Cl-Trp is approximately 3 Å from Nε of the catalytic Lys79.

In chains without Trp or 6-Cl-Trp bound (BorH/FAD + BorH/Trp chains C, D, apo-BorH chains A–D), the density is weaker for the substrate binding lid (α14-α15, Val447-Glu460), suggesting that region becomes more highly ordered in the presence of substrate.

### Binding of Trp, FAD, and FADH_2_ to BorH and AbeH

**Figure 4:**
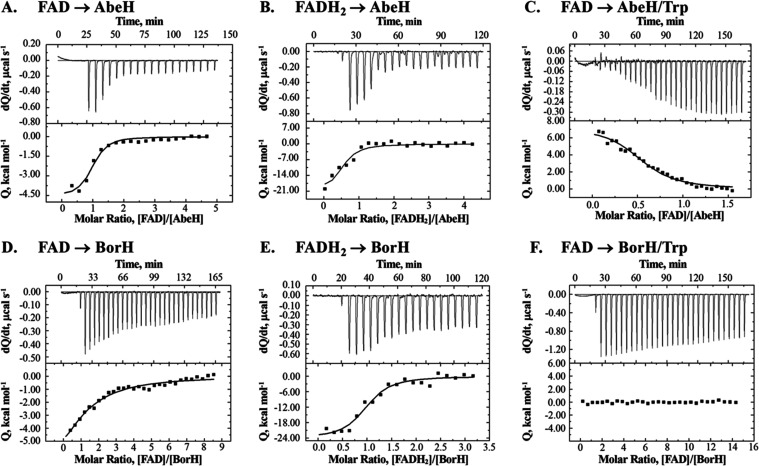
ITC analysis of FAD and FADH_**2**_ binding to AbeH and BorH. The top graph in each panel is the raw differential thermogram of titration with baseline as a solid line, and the bottom graph is the integrated titration curve showing corrected heat for each injection (■) and best fit line (^**__**^). **A**: Titration of FAD into AbeH. **B.** Titration of FADH_2_ into AbeH. **C**: Titration of FAD into AbeH/Trp. Binding was endothermic. **D:** Titration of FAD into BorH. **E** Titration of FADH_2_ into BorH. **F**: Titration of FAD into BorH/Trp (95% saturated). No binding was observed.

**Figure 5:**
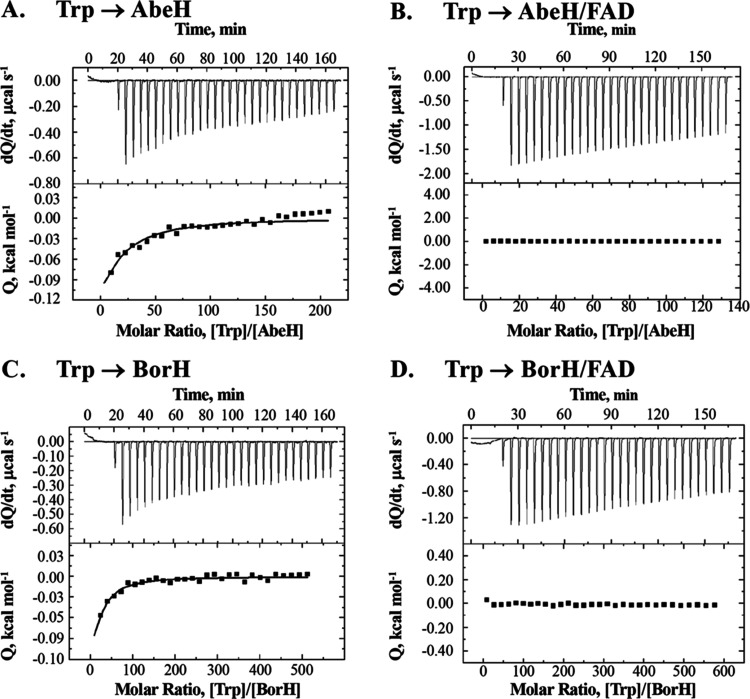
ITC analysis of Trp binding to AbeH and BorH. The top graph in each panel is the raw differential thermogram of titration with baseline as a solid line, and the bottom graph is the integrated titration curve showing corrected heat for each injection (■) and best fit line (^**__**^). **A**: Titration of Trp into AbeH. Binding was only observed at high molar ratio of Trp: AbeH **B**: Titration of Trp into AbeH/FAD. No binding was observed. **C**: Titration of Trp into BorH. Binding was only observed at high molar ratio of Trp: BorH **D**: Titration of Trp into BorH/FAD. No binding was observed.

We used isothermal titration calorimetry to analyze the binding of Trp, FAD, and FADH_2_ to AbeH and BorH (Figures 4–5 and S7–S8; Tables 3 and S3). The apparent *K*_D_ for the AbeH/FAD complex was determined by ITC to be 1.2 μM (Figure 4A), which was consistent with values we independently determined from fluorescence quenching titration experiments using FAD fluorescence (*K*_D_ = 1.6 μM) (Figure S10A) and AbeH intrinsic Trp fluorescence (*K*_D_ = 0.9 μM) (Figure S9B). FAD binds to BorH with approximately 10-fold lower affinity than it binds to AbeH, with *K*_D_ for the BorH/FAD complex of 13 μM by ITC (Figure 4D) and 9.1 μM by quenching of BorH intrinsic Trp fluorescence upon FAD titration (Figure S9A). FADH_2_ binds with higher affinity than FAD to both AbeH (Figure 4B) and BorH (Figure 4E), with *K*_D_ = 0.1 μM for AbeH/FADH_2_ and *K*_D_ = 0.2 μM for BorH/FADH_2_ as determined by ITC.

**Table 3:** *K*_D_ Values Determined for FAD, FADH_2_, and Trp Binding to AbeH and BorH^a^

ligand	protein/preincubated complex	method	*K*_D_ (μM)
FAD	AbeH	ITC	1.2 ± 0.4
	AbeH	FAD fluorescence	1.6 ± 0.4
	AbeH	AbeH intrinsic Trp fluorescence	1.0 ± 0.1
	AbeH/Trp	ITC	6.3 ± 2.4
	AbeH/Trp	FAD fluorescence	7.7 ± 2.2
	BorH	ITC	13 ± 3.3
	BorH	BorH intrinsic Trp fluorescence	9.1 ± 2.4
	BorH/Trp	ITC	no binding
FADH_2_	AbeH	ITC	0.12 ± 0.06
	BorH	ITC	0.21 ± 0.08
Trp	AbeH	ITC	860 ± 140
	AbeH/FAD	ITC	no binding
	BorH	ITC	430 ± 60
	BorH/FAD	ITC	no binding

a Other ITC determined thermodynamic parameters are in Table S3.

When Trp was titrated into either AbeH or BorH, heat of binding had only evolved only at high molar ratios of Trp to protein (Figures 5A,C, S7). Nonlinear regression of ITC data for Trp binding to a one-site model in Origin was fixed to 1 to approximate the values for *K*_A_, Δ*H*, and Δ*S*. The calculated Trp affinities of AbeH and BorH are 500× and 30× lower than the FAD affinities (*K*_D_ = 0.8 mM for Trp/AbeH and 0.4 mM for Trp/BorH).

Since we were unable to capture crystal structures of ternary FDH/FAD/Trp complexes, we tried to detect the formation of ternary complexes by ITC. When AbeH or BorH was preincubated with FAD followed by titration with Trp, there was no evidence of Trp binding by ITC, even at very high molar ratios of Trp to protein (Figure 5B,D), indicating that AbeH/FAD and BorH/FAD complexes are unable to bind Trp.

Titrating FAD into BorH preincubated with Trp also failed to detect any heat of binding, suggesting that BorH/Trp complexes are unable to bind FAD (Figure 4F), mirroring the partitioning observed in the crystal structure. In contrast, titrating FAD into AbeH preincubated with Trp resulted in heat being absorbed rather than released (Figure 4C). The apparent *K*_D_ for FAD binding to AbeH in the presence of Trp determined by ITC is 2–5× higher than in the absence of Trp and is dependent on Trp concentration (*K*_D_ = 2–6 μM, depending on the Trp: AbeH molar ratio; Figure S8). This dependence of apparent FAD affinity on Trp concentration was cross validated by fluorescence quenching titration (Figure S10).

## Discussion

Despite numerous attempts to incorporate Trp by cocrystallization and soaking, no electron density maps generated from diffraction of the orthorhombic AbeH crystals grown in the presence of FAD or the monoclinic AbeH crystals that grew in the absence of FAD revealed density for Trp bound to the substrate binding site. Though Trp alone or 6-Cl-Trp alone could be successfully soaked into the substrate binding site of all four chains of monoclinic apo-BorH crystals, crystals simultaneously soaked in both FAD and Trp resulted in a structure in which FAD and Trp partitioned into different chains in the asymmetric unit, producing two BorH/Trp binary complexes and two BorH/FAD binary complexes. Our results were consistent with an inability to form ternary complexes. Though crystal structures of ternary FDH/flavin/Trp complexes have been reported for RebH (2OA1B),^68^ PrnA (2AQJA),^25^ and PyrH (2WETB),^30^ partitioning similar to our results has been seen previously for PyrH,^30^ RebH^68^ and Thal^67^ crystals, and FAD soaking difficulties have been reported for Xcc4156 apo-crystals.^71^

Negative coupling of FAD and Trp binding was previously proposed for Thal based on similar crystallographic partitioning.^67^ To investigate whether this was a crystallographic artifact or whether there was negative coupling occurring in solution, we carried out a series of thermodynamic binding analysis of FAD and Trp to AbeH and BorH. The ITC experiments were designed to test whether FAD and Trp could bind to AbeH or BorH simultaneously (ternary complex), or whether the two binding sites, despite being ∼10 Å apart from one another, are negatively coupled (only one of the two sites can be occupied at a time).^72^

The ITC results were consistent with our crystallographic observations. Heat was evolved indicating the formation of binary complexes between BorH or AbeH with FAD (Figure 4A,D),FADH_2_ (with μM affinity) (Figure 4B,E), and Trp (with mM affinity) (Figure 5A,C). We saw no evidence of binding of Trp to preincubated AbeH/FAD (Figure 5B) or BorH/FAD (Figure 5D) or binding of FAD to preincubated BorH/Trp (Figure 4F). Unexpectedly, titration of FAD into AbeH/Trp was endothermic and the apparent *K*_D_ for FAD binding to AbeH increased with increasing Trp concentration (Figure 4C).

Binding of FAD to AbeH is enthalpically driven but binding of FAD to AbeH/Trp is entropically driven (Table S3). This may indicate that FAD displaces Trp from the AbeH/Trp complex rather than a ternary complex being formed. It also may reflect the fact that the Trp binding site “lid” in AbeH and other members of its clade (Figure S11) is disordered in the absence of bound Trp, so ejection of Trp from the binding site upon FAD binding could increase the conformational entropy of AbeH.

The different behavior observed upon titration of FAD into AbeH/Trp compared to BorH/Trp could be due to BorH’s 10-fold lower affinity for FAD and 2-fold higher affinity for Trp compared to AbeH, meaning that the thermodynamic driving force for Trp displacement by FAD (FDH/Trp → FDH/FAD) is significantly higher in AbeH than BorH. The structural basis for the negative coupling may also be different in BorH and AbeH. The topology of the Trp binding site is significantly different in AbeH and BorH. Phylogenetic analysis places AbeH and BorH in two different clades (Figure S11). The AbeH clade includes the Trp-5-halogenases AbeH and PyrH^30^ and the Trp-6-halogenases Th-Hal,^24^ SttH,^24^ and Tar14.^69^ The BorH clade includes the Trp-6-halogenases BorH and Thal^31^ and the Trp-7-halogenases PrnA^25^ and RebH.^68^ These two clades have insertions and deletions in the pyramidal subdomain that alter the Trp binding site (Figures S12, S13). The Trp binding site “lid” in AbeH/PyrH is between α6 and β9 (residues 152–167). In the BorH/Thal clade, the connection between these two secondary structural elements is shorter with a different conformation and does not form part of the Trp binding site. In the BorH/Thal clade, the “lid” is part of an extended insertion between α14-α17 in BorH (Val447-Glu460), which contains two short perpendicular α-helices and is missing in the AbeH/PyrH clade (Figure S13). In both clades, the “lid” is less ordered in the absence of Trp, but the different topology of the two subfamilies means that conformational changes in the “lid” may be coupled to different regions of the proteins.

Previous models for FDH negative coupling focus on the opening and closing of the flavin binding loop (Ser36-Glu46 in AbeH, Ala39-Glu49 in BorH), and its consequences on residues immediately following the loop. The flavin binding loop is usually closed in structures with FAD or FADH_2_ bound and open (or absent due to weak or missing electron density) in the absence of flavin. At the C-terminus of the flavin binding loop is Glu46(AbeH)/Glu49(BorH), the Cα of which is a pivot point about which the Glu side chain and peptide backbone exchange positions when the flavin binding loop moves from open (Glu side chain points toward vacant isoalloxazine binding site) to closed (Glu side chain flips out of binding site to make room for isoalloxazine).

Residues immediately C-terminal to the flavin binding loop (Phe49/Ser50 in AbeH/PyrH, Val52/Pro53 in BorH/Thal) have been implicated in the inhibition of Trp binding, as they move into the Trp binding site when the flavin binding loop closes and move out when the loop opens, due to backbone shifts caused by the Glu46/Glu49 flip. This region is variously described as the Trp “gate”^29^ and the “Trp 1 region”.^62^ Closing of the Trp “gate” can be seen when comparing the crystal structures of AbeH/FAD (8FOV) and PyrH/Trp(2WEUA).^30^ PyrH is the closest AbeH homolog (62% identity) that has been solved in a complex with Trp, and since it is also a Trp-5-FDH like AbeH and all the residues contacting Trp in PyrH are conserved in AbeH, it is a reasonable proxy for the AbeH/Trp structure that has as yet eluded us. The unoccupied Trp binding sites in AbeH/FAD and apo-AbeH superimpose perfectly with the unoccupied Trp binding site in PyrH/FAD (2WETC).^30^ When these structures are compared to PyrH/Trp(2WEUA), it can be seen that closing of the flavin binding loop in AbeH/FAD causes the Glu46 switch to flip, closing the Trp “gate” by pushing Phe49 and Ser50 into the Trp binding pocket by a backbone translation of ∼3 Å and side chain rotation, obstructing the space occupied by the Trp β and α carbons and carboxylate in the PyrH/Trp structure (Figure S14).

The “lid”, formed by the loop between 3_10_ helix 3 and strand β9 (148–166), is ordered from 152–166 when occupied (in PyrH/Trp)^30^ and contains Gln160 and Gln164, both of which form H-bonds with the carboxylate of Trp. In both AbeH structures (with the Trp binding site unoccupied), this loop is disordered from 148–160, and 161–166 have moved away from the Trp binding site, with the Gln164 side chain approximately 8 Å away from its position in PyrH/Trp and facing away from the binding site. Structures of PyrH without Trp bound^30^ have the same conformation of the 161–166 portion of the “lid” as the two AbeH structures.

A potential mechanism for negative coupling in AbeH is that binding of FAD or FADH_2_ closes the flavin binding loop and causes Glu46 to flip from in to out, which in turn closes the “gate” by pushing Ser49/Phe50 into the binding site, displacing/obstructing Trp and allowing the “lid” to become open and partially disordered (Figure S14). The structures of AbeH/FAD and PyrH/FAD (flavin binding loop closed, Glu out, gate closed, lid open) and PyrH/Trp (flavin binding loop open, Glu in, gate open, lid closed) are consistent with this mechanism. Three Trp-6-FDH crystal structures from this clade without Trp bound are also consistent with this model (Table S4): Tar14/FAD^69^ (6NSD, 68% identical to AbeH), SttH/FAD^24^ (5HY5, 68%), and apo-Th-Hal^24^ (5LV9, 67%). FAD-bound SttH and Tar14 have closed flavin binding loops, Glu out, and closed gates, while apo-Th-Hal has an open flavin loop, Glu in, and open gate. Tar14, which crystallized in the presence of FAD and Trp, has FAD bound (but not Trp) with a closed flavin binding loop, Glu out, and closed gate, but the lid is closed.

Not all crystal structures are consistent with this simple model (Table S4). In apo-AbeH, even though the flavin binding loop is open/disordered and Glu46 points inward, the gate is closed, and the lid is open. The closed gate explains our inability to soak Trp into this crystal form, but it is unclear why the gate remains closed even though the flavin binding loop is open. A PyrH/FAD/Trp ternary complex^30^ was observed in 2WETB, which has a closed flavin binding loop with an open gate and closed lid, accommodating both FAD and Trp. The electron density for the isoalloxazine and ribityl groups of FAD is much weaker than the adenosine in this chain, suggesting that full occupancy of the isoalloxazine requires a closed Trp gate.

A recent analysis of PyrH in the presence and absence of halide, Trp, 5-Cl-Trp, and FAD/FADH_2_ by Fourier transform infrared (FTIR) difference spectroscopy has provided additional evidence of coupling between the flavin and Trp binding sites.^73^ The authors observed changes in α-helical content associated with oxidation/reduction of flavin that they attributed to dynamic changes in the Trp binding site, which would be consistent with the opening and closing of the lid as described above. Their data also suggests an increase in antiparallel β-sheet content upon photoreduction of bound flavin in the absence of bound Trp, and they propose that this could be related to a short extension of β18. Though we see no evidence for changes in the corresponding β-strand when comparing apo-AbeH and AbeH/FAD, it is notable that their proposed structural change in PyrH is immediately preceding a region (261–265) which is disordered in four of our six AbeH chains. The change in β-stand content detected by FTIR could also be due to the flipping of the Glu46 switch; Gly45-Glu46-Ala47 form a short antiparallel β-strand when the switch is in (apo-AbeH) but not when the switch is out (AbeH/FAD).

Val52/Pro53 in BorH correspond to AbeH Phe49/Ser50 (the Trp gate), and a similar mechanism coupling flavin binding loop closure to blockage of the Trp binding site by the gate has been previously proposed for Thal.^67^ Val/Pro are less bulky than Phe/Ser and the displacement of these residues into the Trp binding site in Thal are smaller, moving about 1 Å rather than 3 Å in the AbeH/PyrH clade. This apparently greater amplitude movement into the Trp binding site in the AbeH/PyrH clade is consistent with our ITC data, which suggested that binding of FAD can eject Trp from AbeH/Trp but not from BorH/Trp. Our BorH structures do not contribute much to the understanding of negative coupling in this clade, because they were all crystallized as apo-BorH and had ligands soaked in, and they all have disordered flavin binding loops and open gates, whether or not FAD is bound. In our BorH structures as well as published Thal^31,67^ structures, the lid has weaker density or is completely disordered when the substrate binding site is vacant.

Crystal packing explains the lack of FAD bound to chains A and B of 8TTI. An A/B contact between dimers in adjacent ASU’s sterically hinders access to the FAD binding site and restricts the possible conformations of the flavin binding loop. This crystal contact is incompatible with both the open and closed flavin binding loop conformations observed in other structures, and there is no room for the AMP group of FAD to be accommodated without steric clashes (Figure S15). Since apo-BorH crystals were soaked with FAD and Trp to solve this structure, this contact prevented binding of FAD to chains A and B. The C/D interface between dimers in the asymmetric unit also involves interactions between the face of BorH containing the FAD binding site, but all three conformations of the flavin loop seen in Thal structures (open, loose, closed) can be modeled at this interface with no steric clashes (Figure S15). However, this close contact between chains C and D still apparently affects the dynamics of the flavin binding loop, as evidenced by the lack of density for the loop and the weaker density for the isoalloxazine in chains C and D. This suggests that the flavin binding loop is not staying “latched” and remains mobile, allowing movement of the isoalloxazine and ribityl groups in and out of the binding groove while the AMP portion stays fixed. The weak density following the missing flavin binding loop allows the Glu49 switch to be modeled equally well in both the in and out configurations in BorH/FAD. The fact that the gate (Val52/Pro53) remains in the open position even in the FAD-bound chains is consistent with the increased mobility of the isoalloxazine and the disorder of the flavin binding loop.

Since the gate is open, it is not completely clear why Trp is prevented from binding to the FAD-bound chains (8TTI chains C and D). Superposition of those chains with the Trp bound chains (A and B) show no differences in the position of the gate, though the density is weaker and noisier for residues in the lid in chains C and D compared to A and B. There are some small positional differences between aromatic residues lining the Trp binding site in chains C and D compared to A and B (Tyr453, Tyr454, Trp465, Phe464), slightly shrinking the Trp binding site in the FAD-bound chains. A network of side chains postulated to be involved in the negative coupling of FAD and Trp in Thal (Tyr362, Asn54, Glu360 in BorH) also show some small positional shifts when comparing the BorH/FAD chains with the BorH/Trp chains, but there are no differences observed in the hydrogen bonding networks.

BorH and AbeH both have higher affinity for FADH_2_ than for FAD (BorH/FAD *K*_D_ = 13 μM, BorH/FADH_2_*K*_D_ = 0.2 μM, AbeH/FAD *K*_D_ = 1.2 μM, AbeH/FADH_2_*K*_D_ = 0.1 μM), while their flavin reductase counterparts BorF and AbeF have higher affinity for FAD than FADH_2_. (BorF/FAD *K*_D_ = 0.1 μM, BorF/FADH_2_*K*_D_ = 0.8 μM, AbeF/FAD *K*_D_ = 0.8 μM, AbeF/FADH_2_*K*_D_ = 1.5 μM).^66^ This is consistent with the directionality of the catalytic cycle (FADH_2_ is the substrate for BorH/AbeH, while FAD is the substrate for BorF/AbeF) and may promote efficient flavin transfer between subunits as has been suggested for other two-component diffusible-flavin oxidoreductase systems.^26^ The intracellular concentration of free FAD/FADH_2_ in bacteria is approximately 8 μM,^74^ suggesting that *in vivo* BorH and AbeH are operating well below saturation. This is consistent with the observation that enzymes from secondary metabolic pathways are as a group less catalytically efficient than those catalyzing reactions of primary metabolism, reflecting the comparatively low selective pressure for their optimization through evolution.^75^ From a practical point of view, is this a fatal flaw that will limit the utility of FDHs for *in vitro* biosynthetic applications, or does it represent an untapped potential for dramatic improvement in catalytic efficiency through rational engineering and directed evolution?

Our ITC data suggest that the negative coupling previously observed in Thal^67^ is present not only in BorH (which is closely related to Thal), but also in the more distantly related AbeH, suggesting it may be a general feature of FDHs and provides an explanation for our inability to capture a ternary complex by crystallography. The negative coupling limits catalytic efficiency, since having one of the two binding sites (flavin or substrate) unoccupied at any given time means that the tunnel connecting the two sites will always be open to bulk solvent at one end or the other, allowing leakage of HOCl/HOBr into solution, leading to decoupling of the redox reaction from the halogenation reaction, and potentially leading to unwanted side reactions and protein oxidation. The rate of NADH consumption exceeds the rate of halogenated product formation, which is why a 5-fold excess of NADH is required to drive Trp chlorination to completion, though this could be due to decoupling between the FDH and FR due to side reactions of diffusible FADH_2_ in solution as well as potential HOCl/HOBr leakage due to negative coupling in the FDH. HOBr leakage has been previously shown for Thal^28^ and tunnel modification has been shown to improve retention of HOBr.^29^ It also limits turnover by making it impossible to carry out halogenation reactions at saturating concentrations of flavin, as unoccupied flavin binding sites are apparently necessary for binding of Trp. If the structural mechanism for negative coupling could be more completely elucidated, it may be possible to engineer mutations that would “break” the coupling and allow Trp to bind before dissociation of FAD, allowing reactions to be carried out at higher flavin concentrations without interfering with Trp binding. This presents a previously unexplored strategy for improving FDH catalytic efficiency, but future work is required to more fully characterize the structural basis for the coupling.

## Conclusions

Purified AbeH chlorinates and brominates Trp at C5 and can halogenate other substrates with indole, phenyl, or quinoline groups, complementing BorH which halogenates Trp at C6 and halogenates an overlapping but nonidentical group of the other tested substrates. Binding studies indicate that both AbeH and BorH bind FADH_2_ more tightly than FAD and bind Trp with very low affinity. The results of crystallographic and ITC studies are consistent with an inability for either AbeH or BorH to form ternary complexes with both FAD/FADH_2_ and Trp. Coupling of the closing of the flavin binding loop and the consequent flipping of the Glu49 switch and closing of the Trp gate explain the inability of FAD and Trp to form a ternary complex with AbeH and also provides a structural explanation by which FAD binding can displace Trp from AbeH/Trp in an entropy-enthalpy compensated manner as our ITC data suggests. If BorH operates like its close homolog Thal, the smaller movement of the Trp gate previously observed in Thal structures upon the flavin binding loop closing could be the explanation for why FAD did not seem to displace Trp from BorH/Trp in our ITC experiments, along with the higher Trp and lower FAD affinity observed in our binding studies.

## Methods

### Protein Expression and Purification

The *abeH* gene was amplified by PCR from a cosmid containing the BE-54017 gene cluster (generously provided by Sean Brady, Rockefeller University) and inserted into expression vector pLIC-HTA using ligation-independent cloning to create an expression construct with an N-terminal His_6_-tag and a TEV protease cleavage site. AbeH was overexpressed in Rosetta(DE3) *E. coli* competent cells (Novagen) grown in Terrific Broth (RPI Corporation) supplemented with 100 μg/mL ampicillin and 30 μg/mL chloramphenicol and protein expression was induced with 0.1 mM IPTG (isopropyl β-d-thiogalactopyranoside; Gold Biosciences) at 16 °C. Cells were harvested 17–20 h after induction and stored at −80 °C. Cell pellets were resuspended in lysis buffer (50 mM Tris-HCl pH 8.2, 500 mM NaCl, and 10 mM imidazole) and subjected to three freeze–thaw cycles followed by sonication and clarification by centrifugation. AbeH was captured from clarified lysates using immobilized metal affinity chromatography and further purified by anion exchange and size exclusion chromatography. Purified AbeH in SEC buffer (20 mM HEPES pH 7.2 and 35 mM sodium citrate) plus 20% (v/v) glycerol was concentrated to 5 mg/mL, flash-frozen, and stored at −80 °C. Protein concentrations were determined by absorbance at 280 nm with calculated extinction coefficients. Protein purity and homogeneity was assessed by sodium dodecyl sulfate polyacrylamide gel electrophoresis (SDS-PAGE) and gel filtration chromatography (Superdex 200 16/60). BorH, BorF, and AbeF were purified as previously described.^23,66^ Proteins used for ITC binding experiments with FADH_2_ were purified in the absence of halide using 20 mM HEPES pH 7.5 100 mM sodium citrate instead of Tris-HCl/NaCl in all buffers (lysis, affinity chromatography, and SEC).

### Analysis of *In Vitro* Halogenation of Trp by AbeH Using HPLC and MS

*In vitro* halogenation reactions contained 7 μM AbeH, 13 μM holo-AbeF (∼20% saturated with FAD), 2.5 mM NADH, 0.5 mM Trp, 50 mM NaCl or NaBr in 10 mM Tris-HCl pH 8.2 in a final volume of 100 μL. Reactions were initiated by addition of NADH and incubated with 200 rpm shaking at 37 °C for 1 h. Reactions were stopped by heating to 95 °C for 10 min followed by centrifugation at 10,000*g* for 5 min. Reaction mixtures were analyzed by RP-HPLC using a Kromasil C18 RP-analytical column (100 Å pore size, 4.6 × 250 mm) mounted on a Waters 2695 HPLC. Separation used a 14 min gradient from 5% B to 100% B (Solvent A = H_2_O/CH_3_CN 95:5, 0.1% TFA; Solvent B = H_2_O/CH_3_CN 5/95, 0.1% TFA) with flow rate 0.6 mL/min. Trp, 5-Cl-Trp, and 5-Br-Trp peaks were monitored with absorbance at 280 nm and retention times and yields were analyzed with using retention times and peak areas from standard curves of authentic standards of Trp (ACROS Organics), 5-Cl-Trp (Santa Cruz Biotechnology) and 5-Br-Trp (Santa Cruz Biotechnology). RP-HPLC purified 5-Cl-Trp and 5-Br-Trp peaks from *in vitro* halogenation reactions were analyzed on a Waters SYNAPT HDMS mass spectrometer using positive ion-mode ESI-MS with leucine enkephalin as internal standard.

To determine the Cl-Trp regioisomer produced by AbeH, 2 mg Trp was chlorinated by incubating 7 μM AbeH, 13 μM holo-AbeF, 2.5 mM NADH, 500 μM Trp, 100 mM NaCl, 10 mM Tris-HCl pH 8.2 for 20 min at 37 °C, after which the reaction was stopped by heating to 95 °C for 10 min and precipitated proteins were pelleted by centrifugation at 10,000*g* for 5 min. Clarified reaction mixtures were concentrated on a silica gel flash column before RP-HPLC purification. The product was dried by rotary evaporation and lyophilization and dissolved in D_2_O for ^1^H NMR analysis using a 600 MHz Bruker Avance III.

Time-course experiments were done using 1.7 μM AbeH, 3.3 μM holo-AbeF, 500 μM Trp, 2.5 mM NADH in 10 mM Na/KPO_4_ pH 8.2 with either 50 mM NaCl or 100 mM NaBr. Reactions were incubated for 5 min at 37 °C and NADH was added to start the reactions. Aliquots of 100 μL were quenched and analyzed by RP-HPLC at various time points. Unreacted substrate and product concentrations were calculated using peak areas and standard curves of Trp, 5-Cl-Trp, and 5-Br-Trp.

### Halogenation of Non-Trp Substrates by AbeH and BorH

To test the ability of BorH and AbeH to chlorinate and brominate other substrates, 100 μL reactions containing 5 μM AbeH or BorH, 10 μM of holo-AbeF or holo-BorF, 100 mM NaCl or NaBr, 2.5 mM NADH, and 0.5 mM substrate in HEPES pH 8.0 were incubated for 12 h at 37 °C with shaking at 230 rpm. All substrates tested are shown in Figures S5 and S6, substrates that produced halogenated products are also shown in Tables 1 and S1. The reactions were stopped by heating at 95 °C for 5 min and centrifuged at 12,000*g* for 5 min. Clarified reaction mixtures were analyzed using RP-HPLC on a Kromasil C18 column (100 Å pore size, 4.6 × 250 mm) mounted on a Waters 2695 HPLC. The gradient was as follows: 0–7.5 min, 5–50% B; 7.5–14 min, 50–100%; 14–25 min, 100% B; 25–40 min 100–5% B with flow rate 0.6 mL/min (Solvent A = H_2_O/CH_3_CN 95:5, 0.1% TFA; Solvent B = H_2_O/CH_3_CN 5/95, 0.1% TFA). Substrates and products were detected by monitoring UV absorbance at the λ_max_ for each substrate. Product peak fractions were analyzed using ESI-MS on a SYNAPT HDMS mass spectrometer in positive ion mode with leucine enkephalin internal standard.

### Crystallization and Structure Determination

AbeH/FAD complex for crystallization was prepared by incubating 50 μM AbeH (in 20 mM HEPES pH 7.2 and 35 mM sodium citrate with 2.5 mM FAD for 20 h at 4 °C. AbeH/FAD crystals grew overnight in drops containing a 1:1 ratio of AbeH/FAD with reservoir solution (0.1 M Bis-Tris pH 6.2, 0.2 M magnesium acetate, 11% (v/v) PEG 10,000) equilibrated against 1 mL reservoir solution. AbeH/FAD crystals were cryoprotected using reservoir solution supplemented with 20% (v/v) glycerol and flash-frozen in liquid nitrogen for X-ray data collection. To prepare AbeH/FAD/Trp ternary complex crystals by soaking, some AbeH/FAD cocrystals were soaked with Trp (2.5–50 mM) for variable lengths of time (5 min to 84 h) before cryoprotection and freezing.

Apo-AbeH crystals were grown using the hanging drop vapor diffusion method at room temperature with drops containing 2 μL of protein (40 μM AbeH/20 mM HEPES pH 7.5/350 mM NaCl) and 2 μL of reservoir solution (100 mM Bis-tris propane pH 7.0, 150 mM MgSO_4_, 25% (v/v) PEG 3350) equilibrated against 500 μL of reservoir solution. Apo-AbeH crystals were cryoprotected in reservoir solution containing 20% (v/v) 2,3-butanediol for 20 min, and flash-frozen in liquid nitrogen for X-ray data collection. To prepare AbeH/Trp ternary complex crystals by soaking, some apo-AbeH crystals were soaked with reservoir solution containing 20% (v/v) 2,3-butanediol and 50 mM Trp for 50 min before freezing.

AbeH/FAD X-ray diffraction data were collected at the Life Sciences Collaborative Access Team beamline 21-ID-F at the Advanced Photon Source (Argonne National Laboratory) with a Rayonix MX 300 detector using the oscillation method (0.5° per frame).^76^ Data processing used iMOSFLM for indexing and integration, and Pointless, Aimless, Scala and Truncate for scaling, merging, and structure factor generation.^77,78^ Molecular replacement was done using Phaser-MR in *Phenix* using 5LV9 (Chain B) (Th-Hal, 66.1% sequence identity to AbeH) as a search model.^24,79,80^ The initial AbeH model was built using AutoBuild, followed with iterative cycles of manual model building in Coot and refinement (rigid body, XYZ, and group B-factors) with Phenix.Refine until both chains were modeled. The FAD binding site was identified visually and using phenix.Ligandfit. Refinement of the AbeH/FAD complex was carried out using Phenix.Refine (XYZ, individual B-factors, occupancies, and TLS parameters). Model validation was performed routinely using MolProbity^81^ after each refinement and comprehensive model validation was done in *Phenix* GUI. Apo-AbeH X-ray diffraction data were collected at beamline 21-ID-G and processed using the same method as for AbeH/FAD. The apo-AbeH crystal structure was solved by Phaser-MR using chain B of the AbeH/FAD model. Refinement and model building was performed as described for the AbeH/FAD structure.

Crystals of BorH were grown, soaked, and cryoprotected as described previously for the BorH/Trp crystal structure.^23^ For this study, apo-BorH crystals, BorH crystals soaked in 6-Cl-Trp, and BorH crystals soaked in both FAD and Trp were frozen and analyzed. X-ray diffraction data were collected at 100 K on a Dectris Eiger X9M detector using the oscillation method (0.25° per frame) at the Life Sciences Collaborative Access Team beamline 21-ID-D at the Advanced Photon Source (Argonne National Laboratory). Data were processed in the same manner as described for AbeH and structures were determined by molecular replacement using Phaser-MR with chain A of the BorH/Trp structure (6UL2.pdb) as a search model. Refinement and structural validation were carried out as described earlier for AbeH.

#### Analysis of Trp, FAD, and FADH_2_ Binding by Isothermal Titration Calorimetry

ITC experiments were performed using a VP-ITC isothermal titration calorimeter (MicroCal) in 20 mM HEPES pH 7.5, 350 mM NaCl, 1 mM ethylenediamine tetraacetic acid (EDTA) and 15% (v/v) glycerol. Solutions of AbeH, BorH, FAD (Chem-Implex), and Trp (Acros Organics) were prepared in the same buffer and degassed under vacuum at 30 °C for 20 min before loading in the syringe or cell. To analyze formation of binary complexes, AbeH or BorH was placed inside the cell, and ligand (FAD or Trp) was injected. For ternary complex titration, one ligand was preincubated with the macromolecule for 30 min at 30 °C to form binary complexes of AbeH/FAD, AbeH/Trp, BorH/FAD, and BorH/Trp before titrating with the second ligand. To test FADH_2_ binding to AbeH and BorH, FAD was reduced to FADH_2_ using sodium dithionite and stored under N_2_. Formation of FADH_2_ by sodium dithionite was verified using absorbance and fluorescence spectrophotometry.

ITC experiments were performed with reference power set to 10 μcal/s and Milli-Q water in the reference cell. All experiments were done with a pretitration delay of 20 min, a 5 min interval between injections, and a filter period of 2 s in high feedback gain mode. All experiments were performed at 310 rpm to mix the components in cell and titrant. All other VP-ITC instrumental parameters were set to default settings. Heats of dilution for all titrants were measured by titrating ligand into buffer for binary complex titrations, and by titrating ligand into a solution containing the other ligand but no protein for ternary complex titration.

The following titrations were carried out:

1.2 mM FAD (2 μL/1 and 5 μL/19 injections) into 18 μM AbeH (Figure 4A)

0.12 mM FADH_2_ (2 μL/1 and 15 μL/19 injections) into 7 μM AbeH in the presence of sodium dithionite (Figure 4B)

190 μM FAD (2 μL/1 and 15 μL/29 injections) into 28 μM AbeH preincubated with 16 mM Trp (Figure 4C)

190 μM FAD (2 μL/1 and 15 μL/29 injections) into 28 μM AbeH preincubated with 28 mM Trp (Figure S8)

0.3 mM FAD (2 μL/1 and 10 μL/29 injections) into 8 μM BorH (Figure 4D)

43 μM FADH_2_ (2 μL/1 and 15 μL/19 injections) into 3 μM BorH in the presence of sodium dithionite (Figure 4E)

0.63 mM FAD (2 μL/1 and 10 μL/29 injections) into 10 μM BorH preincubated with 18 mM Trp (Figure 4F)

1.4 mM Trp (3 μL/1 and 10 μL/29 injections) into 43 μM AbeH (Figure S7)

16 mM Trp (2 μL/1 and 10 μL/29 injections) into 18 μM AbeH (Figure 5A)

27 mM Trp (2 μL/1 and 10 μL/29 injections) into 48 μM AbeH preincubated with 3.3 mM FAD (Figure 5B)

21 mM Trp (2 μL/1 and 10 μL/29 injections) into 8 μM BorH (Figure 5D)

21 mM Trp (2 μL/1 and 10 μL/29 injections) into 8 μM BorH preincubated with 5 mM FAD (Figure 5E)

Titrations were analyzed by manually integrating each injection, subtracting the heat of dilution, and carrying out nonlinear least-squares fit of the calorimetric binding data to a one-site model using Origin 7.0 (Origin Lab). Trp binding experiments were subtracted by point-by-point heat of dilution, and FAD and FADH_2_ binding experiments were subtracted using the mean heat of the last three injections. For the Trp binding data, *n* was fixed to 1 based on expected stoichiometry of 1:1 based on FDH crystal structures and as proposed for lower c-value titrations in the literature.^23,25,82^ The other parameters were not constrained during fitting.

#### Analysis of Trp and FAD Binding by Fluorescence Spectroscopy

Quenching of FAD fluorescence quenching upon binding to AbeH was measured in the absence and presence of Trp in 20 mM Tris-HCl pH 8.0 and 100 mM NaCl using a Quantmaster 40 fluorimeter (Photon Technologies International). Trp and FAD solutions were prepared in 20 mM Tris-HCl pH 8, and concentrations were determined by absorbance at 280 and 450 nm, respectively. FAD fluorescence was measured using excitation at 450 nm (4 nm slit opening) and emission set at 525 nm (6 nm slit opening) using a fluorescence cuvette to collect 50 scans, which were averaged later during data processing in Microsoft Excel. Experiments were performed in duplicate by adding different concentrations of AbeH into fixed FAD (0.45–0.50 mM) and without or with different fixed Trp (0.3–5.5 mM) concentrations. Binding experiments were performed by mixing the components and incubating for 2 min before data collection by excitation at 450 nm and emission set at 525 nm. Raw fluorescence intensities were corrected for the buffer, Trp, and AbeH background contributions. Quenched fluorescence intensity was plotted vs AbeH concentration and fit to eq 1 using GraphPad Prism 8.0 by nonlinear regression analysis. In eq 1, *F* = fractional saturation, Δ*F* = fluorescence quenching upon AbeH/FAD complex formation (calculated from the difference of FAD fluorescence intensity at different AbeH concentration), Δ*F*_max_ = maximum fluorescence change obtained from fit, [AbeH_FAD_] = FAD-bound AbeH, [AbeH]_T_ = Total AbeH concentration, [FAD]_T_ = Total FAD concentration, *K*_D_ = equilibrium dissociation constant.
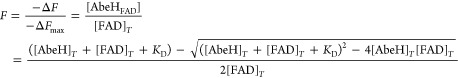
1

#### Intrinsic Tryptophan Fluorescence Spectroscopy

Intrinsic tryptophan fluorescence spectroscopy was used to measure FAD binding to AbeH and BorH in 20 mM HEPES pH 7.7- and 50 mM sodium citrate. FAD was prepared in the same buffer and its concentration was determined spectrophotometrically as described earlier. Fluorescence experiments were performed as described earlier. FAD binding to apo-AbeH or apo-BorH was performed in duplicate with 100 nM fixed AbeH or BorH with variable FAD concentrations up to 30 and 70 μM, respectively. AbeH/FAD binding was monitored using excitation at 295 nm and emission at 328 nm. BorH/FAD binding was monitored using excitation at 290 nm and emission at 326 nm. Inner filter effect corrected fluorescence intensities were plotted against quenched intrinsic tryptophan fluorescence of AbeH and BorH. Data were fitted by nonlinear regression to eq 1 using GraphPad Prism 8.0.

## Data Availability

All X-ray crystal structures have had coordinates and structure factors deposited in the RCSB Protein Data Bank with the following accession numbers: 8FOX (Apo-AbeH), 8FOV (AbeH/FAD), 8TTJ (BorH/6-Cl-Trp), 8TTK (Apo-BorH), 8TTI (BorH/FAD + BorH/Trp).

## Supporting Information Available

AbeH purification chromatograms and gels, RP-HPLC, MS, and NMR data from in vitro AbeH halogenation assays, time-course data for AbeH Trp halogenation, substrate scope for AbeH and BorH, pairwise Cα rmsd values (Å) for BorH and AbeH structures, supporting data for ITC experiments, fluorescence quenching data, ITC-derived thermodynamic parameters for FAD, FADH2, and Trp binding to AbeH and BorH, phylogenetic tree of AbeH and BorH with other FDHs with solved crystal structures, multiple sequence alignment of AbeH and BorH with other FDHs, supplemental figures showing structural differences in Trp binding lid, movement of Trp gate, and crystal packing (PDF)
